# A Dominant Role for the Immunoproteasome in CD8^+^ T Cell Responses to Murine Cytomegalovirus

**DOI:** 10.1371/journal.pone.0014646

**Published:** 2011-02-03

**Authors:** Sarah Hutchinson, Stuart Sims, Geraldine O'Hara, Jon Silk, Uzi Gileadi, Vincenzo Cerundolo, Paul Klenerman

**Affiliations:** 1 Nuffield Department of Clinical Medicine, Peter Medawar Building for Pathogen Research, University of Oxford, Oxford, United Kingdom; 2 Weatherall Institute of Molecular Medicine, Molecular Immunology Group, Nuffield Department of Medicine, John Radcliffe Hospital, Oxford, United Kingdom; Southern Illinois University School of Medicine, United States of America

## Abstract

Murine cytomegalovirus (MCMV) is an important animal model of human cytomegalovirus (HCMV), a β-Herpesvirus that infects the majority of the world's population and causes disease in neonates and immunocompromised adults. CD8^+^ T cells are a major part of the immune response to MCMV and HCMV. Processing of peptides for presentation to CD8^+^ T cells may be critically dependent on the immunoproteasome, expression of which is affected by MCMV. However, the overall importance of the immunoproteasome in the generation of immunodominant peptides from MCMV is not known. We therefore examined the role of the immunoproteasome in stimulation of CD8^+^ T cell responses to MCMV – both conventional memory responses and those undergoing long-term expansion or “inflation”. We infected LMP7^−/−^ and C57BL/6 mice with MCMV or with newly-generated recombinant vaccinia viruses (rVVs) encoding the immunodominant MCMV protein M45 in either full-length or epitope-only minigene form. We analysed CD8^+^ T cell responses using intracellular cytokine stain (ICS) and MHC Class I tetramer staining for a panel of MCMV-derived epitopes. We showed a critical role for immunoproteasome in MCMV affecting all epitopes studied. Interestingly we found that memory “inflating” epitopes demonstrate reduced immunoproteasome dependence compared to non-inflating epitopes. M45-specific responses induced by rVVs remain immunoproteasome-dependent. These results help to define a critical restriction point for CD8^+^ T cell epitopes in natural cytomegalovirus (CMV) infection and potentially in vaccine strategies against this and other viruses.

## Introduction

Human cytomegalovirus (HCMV) is a β-Herpesvirus, a double-stranded DNA virus encoding 160 proteins, which infects most of the world's population [1]. Infection is asymptomatic in immunocompetent hosts; however, virus is not cleared after acute infection and persists lifelong. In immunocompromised hosts, such as those infected with HIV and bone marrow transplant (BMT) recipients, HCMV infection may cause severe disease. Murine cytomegalovirus (MCMV) is a well-characterized animal model for HCMV, reflecting many key aspects of the immunology of human infection [2].

CD8^+^ T cells are important mediators of immune responses to HCMV and MCMV [3], [4], [5], [6], [7]. CD8^+^ T cells in combination with other antiviral mediators limit CMV reactivation in immunocompetent hosts and protect against disease in immunosuppressed hosts. Adoptive transfer of anti-CMV CD8^+^ T cells protects from CMV-induced disease in mouse and man [8], [9]. During the chronic phase of CMV infection CD8^+^ T cell responses to certain epitopes increase in number over time; this phenomenon, called ‘memory inflation’, is characteristic of CMVs [10], [11], [12], [13], [14]. The “superboosting” nature of the immune response can be exploited in CMV-based vaccines to induce long-term protective antiviral CD8^+^ T cell responses [14], [15]. However the mechanism of memory inflation and the criteria that distinguish those responses that inflate compared to those that enter into a classical memory pool are not known.

The CD8^+^ T cell response to virus infection is driven by the presentation of peptide by professional antigen presenting cells (APCs) and infected targets. Generation of the peptide repertoire requires a series of components responsible for cleavage and presentation [16]. The immunoproteasome is one such component, an inducible form of the proteasome thought to be optimised for production of MHC class I ligands [17]. All cells contain constitutive proteasomes and respond to interferon-gamma (IFN-γ) by upregulation of immunoproteasome expression. Immune cells, including professional APCs such as dendritic cells (DCs) and macrophages, also constitutively express immunoproteasomes [18], [19], [20].

The role of the immunoproteasome *in vivo* is not yet defined. Constitutive and immuno-proteasomes produce a different profile of potential CD8^+^ T cell epitopes and epitope precursors from a given polypeptide. In general, immunoproteasome digests contain more potential MHC class I ligands than the constitutive counterparts [21]. However, most known microbial CD8^+^ T cell epitopes are produced by both types of proteasome in cell-free assays and by infected APCs [22]. Mice lacking functional genes for one or two of the three catalytic subunits of the immunoproteasome (LMP7, LMP2 and MECL-1) produce many expected CD8^+^ T cell responses to infection, although the overall impact is not yet clear [19], [23], [24], [25], [26]. One study of lymphocytic choriomeningitis virus (LCMV) infection in LMP7^−/−^ mice, showed no difference in numbers of CD8^+^ T cell responses or difference in viral load and disease [27]. However, the same study showed that in DNA vaccination with LCMV glycoprotein there is enhanced presentation of one epitope (GP276) in the absence of LMP7, a feature noted before in LMP7^−/−^ mice when a rVV expressing the LCMV glycoprotein was used [19]. Recently, it has been shown that LMP7 inhibition using a small molecule inhibitor of LMP7 strongly downregulated the CTL response to LCMV-GP33 and NP396 (but not sub-dominant epitopes) during LCMV infection [28]. Thus, while it is known that specific epitopes can show immunoproteasome dependence, the overall dependence of antiviral T cell responses on immunoproteasomes is not fully defined.

MCMV and HCMV encode numerous immunoevasins which affect components of the MHC class I antigen processing pathway [29]. IFN-γ-mediated upregulation of immunoproteasome expression is normally an important host response to viral infection. However, in HCMV- or MCMV-infected cells it has been shown that upregulation of the immunoproteasome does not occur in response to IFN-γ [30]. In murine cells this effect is mediated by the MCMV protein M27, which prevents signaling through the IFN-γ receptor (IFNGR) by binding to the cellular signalling intermediate STAT2 (previously thought to transduce only signals through the interferon-α receptor (IFNAR)) [31]. It was proposed that if the repertoire of antiviral CD8^+^ T cells was selected on constitutively immunoproteasome-rich professional APCs, such as DCs and macrophages, they might predominantly be specific for epitopes that are better presented by the immunoproteasome [30]. If this were true, M27-mediated suppression of immunoproteasome expression in infected tissue cells might protect infected cells from detection by antiviral CD8^+^ T cells.

We therefore investigated the role of the immunoproteasome in induction of CD8^+^ T cell responses to MCMV. LMP7^−/−^ mice were infected with MCMV or novel recombinant vaccinia viruses (rVVs) expressing MCMV proteins. Antigen-specific CD8^+^ T cell responses to a panel of previously defined peptide epitopes were quantified by *ex vivo* MHC class I tetramer analysis and IFN-γ intracellular cytokine stains (ICS). A key role for the immunoproteasome was revealed in modulating the immunodominant response in acute disease and in a range of other epitopes. This study reveals a major role for the immunoproteasome in determining the CD8^+^ T cell repertoire in MCMV, both in acute infection (7 days) and chronic infection (>50 days).

## Results

### Responses to M45 are not detected in LMP7^−/−^ mice

**Figure 1:**
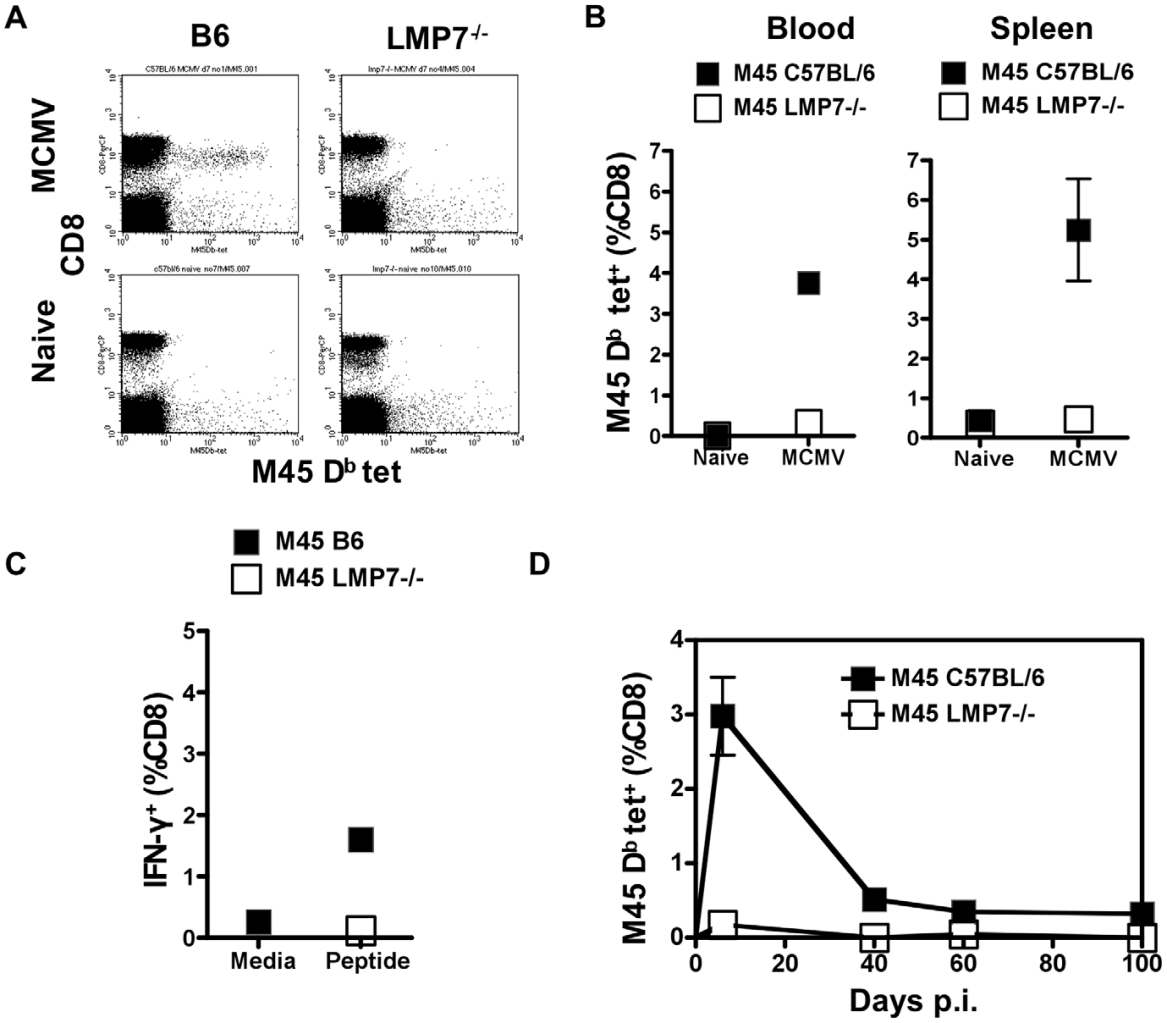
CD8^+^ T cell responses to M45 in LMP7^−/−^ mice infected with MCMV. A. LMP7^−/−^ mice and C57BL/6 control mice were injected intravenously (i.v.) with 1×10^6^ plaque forming units (pfu) MCMV or an equivalent volume of phosphate buffered saline (PBS). Seven days p.i. peripheral blood was sampled by tail bleed. Whole blood was stained with fluorochrome-conjugated soluble tetrameric complexes (M45 H-2 D^b^ ^985^HGIRNASFI^993^) and anti-CD8 antibody, and red blood cells lysed, before analysis by FACS. B. Lymphocytes were prepared from peripheral blood and spleen of LMP7^−/−^ and C57BL/6 control mice 7 days p.i. (n = 5) or PBS treated (blood: n = 2; spleen: n = 4) and subjected to tetramer stain and FACS analysis as above. The plots show the frequency of M45 D^b^-specific cells among CD8^+^ T cells. SEM was less than 0.1%. C. Splenocytes were prepared from spleens of LMP7^−/−^ and C57BL/6 control mice (n = 5) 7 days p.i. and incubated in either media containing synthetic peptide HGIRNASFI or media alone, in the presence of Brefeldin A (BFA). After 5 hours cells were stained for CD8 and intracellular IFN-γ and analysed by FACS. The plots show % CD8^+^ T cells producing IFN-γ in response to peptide or media alone. SEM was less than 0.1%. D. Peripheral blood was sampled from LMP7^−/−^ and C57BL/6 control mice (n = 3) at 7, 40, 60 and 100 days p.i. and subjected to tetramer stains using M45 D^b^ HGIRNASFI tetramer and FACS analysis as for Figure 1A. The plot shows the frequency of M45 D^b^- specific cells among CD8^+^ T cells over a 100 day time course.

In wild type mice, the most dominant CD8^+^ T cell response to MCMV after acute infection (day 7) recognizes the D^b^ restricted epitope M45 HGIRNASFI (hereafter described as “M45”) [32], [33]. We aimed to determine the role of the immunoproteasome in induction of this response by comparing M45-specific responses to MCMV infection in C57BL/6 mice with those in gene-targeted knockout mice lacking a functional gene for the immunoproteasome subunit LMP7 (LMP7^−/−^) backcrossed onto a C57BL/6 background [26]. LMP7^−/−^ and C57BL/6 mice were injected intravenously with MCMV. Seven days later, peripheral blood or splenocytes were prepared and M45-specific responses evaluated by tetramer stain or IFN-γ ICS (Figure 1). Seven days post-infection (p.i.) is the peak of the cellular immune responses to MCMV and conventionally referred to as acute infection.

As expected, 4% of CD8+ T cells from the peripheral blood stained positive with the M45 tetramer in C57BL/6 mice acutely infected with MCMV (Figure 1A). In contrast, no M45-specific CD8+ T cells were detected in the peripheral blood of LMP7-/- mice. To determine whether M45-specific cells were present at another site, splenocytes from LMP7^−/−^ and C57BL/6 acutely infected mice were stained with M45 tetramer. M45-specific splenocytes were below the limit of detection in infected LMP7^−/−^ mice (Figure 1B), compared to substantial responses (5%) detected in C57BL/6 mice. The function of M45-specific CD8^+^ T cells in the spleens of acutely infected LMP7^−/−^ mice was assessed by *ex vivo* IFN-γ ICS. No CD8^+^ T cells from the spleens of infected LMP7^−/−^ mice produced IFN-γ in response to peptide in *ex vivo* IFN-γ ICS (Figure 1C) compared with 1.5% of CD8^+^ T cells in MCMV-infected C57BL/6 controls.

It was possible that the pattern of responses to M45 would be affected by the immunoproteasome in a temporal fashion (i.e. responses may be delayed rather than absent). Using MHC class I tetramer staining to detect M45-specific responses in the blood of LMP7^−/−^ mice up to 100 days p.i., it is apparent that the response to M45 remained below the limit of detection at all time points tested (Figure 1D). In comparison, in C57BL/6 mice, M45-specific cells were maintained over time at a low level (0.4% of CD8^+^ T cells; Figure 1D).

### CD8 responses to m141 are not detected in LMP7^−/−^ mice

**Figure 2:**
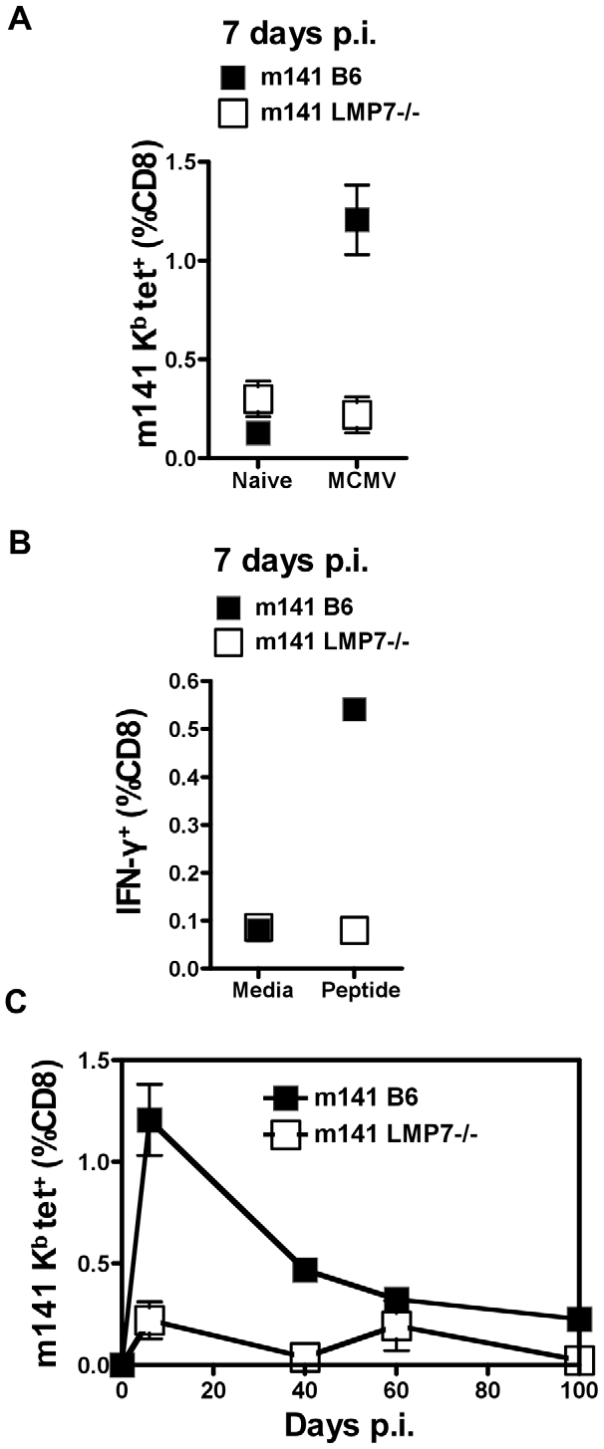
CD8^+^ T cell responses to m141 in LMP7^−/−^ mice infected with MCMV. A. Lymphocytes were prepared from peripheral blood of LMP7^−/−^ and C57BL/6 control mice 7 days p.i. (n = 3) or PBS treated (n = 2) and subjected to tetramer stain and FACS analysis as for Figure 1A, using MCMV m141 tetramer (H-2 K^b^ ^15^VIDASFRL^23^). The plot shows the frequency of m141 K^b^-specific cells among CD8^+^ T cells. B. Splenocytes were prepared from spleens of LMP7^−/−^ and C57BL/6 control mice 7 days p.i. (n = 6), pooled, and incubated in the presence of BFA with either media containing peptide VIDAFSRL or media alone for 5 hours. Cells were stained as in Figure 1C and analysed by FACS. The plot shows % CD8^+^ T cells producing IFN-γ in response to peptide or media alone. C. Peripheral blood was sampled from LMP7^−/−^ and C57BL/6 control mice (n = 3) at 7, 40, 60 and 100 days p.i. and subjected to tetramer stain and FACS analysis as for A. The plot shows the frequency of m141 K^b^-specific cells among CD8^+^ T cells over a 100 day time course.

During acute MCMV infection, a second protein which contains a highly targeted epitope in C57BL/6 mice is m141 [32]. Compared with 1.2% of CD8^+^ T cells in acutely infected C57BL/6 mice (Figure 2A), no m141-specific CD8^+^ T cells were detected in the blood of LMP7^−/−^ mice at 7 days p.i. IFN-γ was not produced in response to m141 peptide by splenocytes from infected LMP7^−/−^ mice, whereas 0.5% of CD8+ T cells from C57BL/6 mice mounted an IFN-γ response to m141 peptide (Figure 2B). In C57BL/6 mice, the number of m141-specific T cells did not inflate over time [11]. The m141 specific responses in the blood of LMP7^−/−^ mice were examined using tetramers between days 7 and 100 p.i. At no point in time were m141-specific CD8+ T cells detected in the peripheral blood of LMP7^−/−^ mice (Figure 2C).

### Responses to M38a and m139 are affected by LMP7 deletion

The M38 and m139 proteins of MCMV are major targets of the CD8^+^ T cell response in C57BL/6 mice. In contrast to M45 and m141 described above, CD8^+^ T cell responses to M38a and m139 both undergo memory inflation. The acute CD8^+^ T cell response to M38a is relatively small and expands only later compared to CD8^+^ T cells specific for m139, which are also relatively abundant during the acute phase of infection [11].

**Figure 3:**
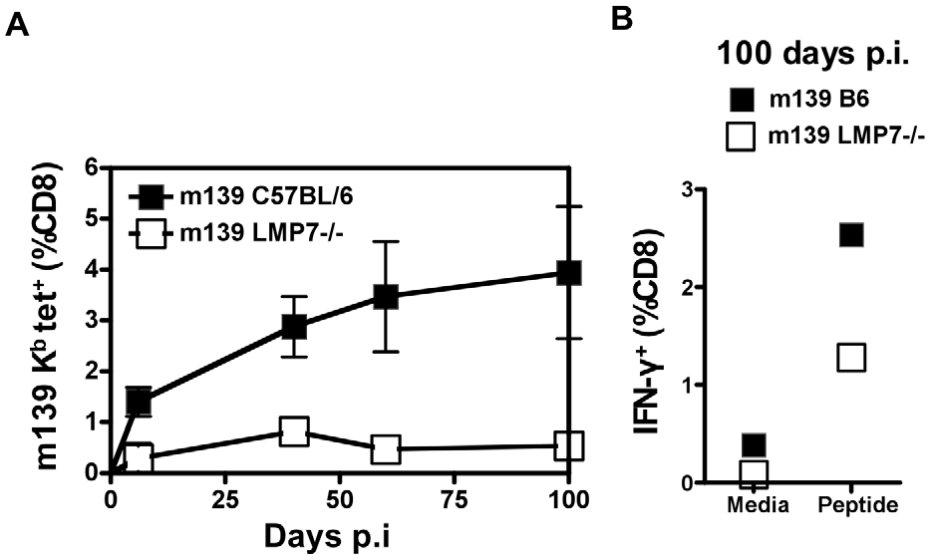
CD8^+^ T cell response to inflating epitope m139 in LMP7^−/−^ mice infected with MCMV. A. Lymphocytes were prepared from the peripheral blood of LMP7^−/−^ and C57BL/6 control mice 7 days p.i. (n = 3) or PBS (n = 2) and subjected to tetramer stain and FACS analysis as for Figure 1A, using MCMV m139 tetramer (H-2 K^b^ ^419^TWYGFCLL^426^). This epitope has been shown to elicit a CD8^+^ T cell response that increases over time, or inflates, in C57BL/6 mice. The plot shows the frequency of m139 K^b^-specific cells among CD8^+^ T cells over a 100 day time course. B. Splenocytes were prepared from spleens of LMP7^−/−^ and C57BL/6 control mice 7 days p.i. (n = 3) and incubated in the presence of BFA with either media containing synthetic peptide TWYGFCLL or media alone for 5 hours. Cells were stained for surface CD8 and intracellular IFN-γ as in Figure 1C and analysed by FACS. The plot shows % CD8^+^ T cells producing IFN-γ in response to peptide or media alone.

Peripheral blood of MCMV-infected LMP7^−/−^ and C57BL/6 mice was assayed for m139-specific CD8^+^ T cells over a 100-day time course (Figure 3A). The CD8^+^ T cell response to m139 in acute infection was reduced (0.3%) relative to the wild type response (1.5%), but remained detectable. The difference was greater in chronic infection; the m139-specific response in C57BL/6 mice increased to 3.5% by 100 days p.i., whereas the m139-specific response of LMP7^−/−^ mice remained static over time (Figure 3A). The same pattern was evident in spleens. IFN-γ-secreting m139-specific CD8^+^ T cells were also detected in the spleens of LMP7^−/−^ mice 100 days p.i. by ICS (Figure 3B), but at a lower frequency than in C57BL/6 controls (1.2% vs 2.5%; Figure 3B).

**Figure 4:**
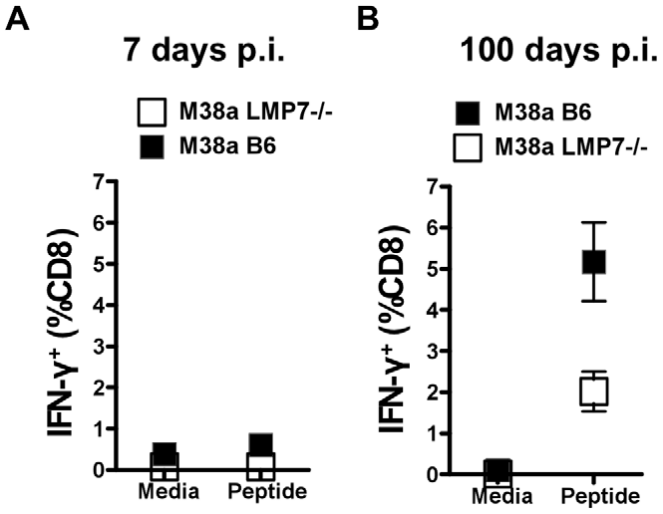
CD8^+^ T cell responses to inflating epitope M38 in LMP7^−/−^ mice infected with MCMV. A. Splenocytes were prepared from spleens of LMP7^−/−^ and C57BL/6 control mice 7 days p.i.(n = 6), pooled, and incubated in the presence of BFA with either media containing peptide SSPPMFRV (M38 H-2 K^b^ ^316^SSPPMFRV^325^) or media alone for 5 hours. Cells were stained for surface CD8 and intracellular IFN-γ as in Figure 1C and analyzed by FACS. The plots show % CD8^+^ T cells producing IFN-γ in response to peptide or media alone. B. Splenocytes were prepared from spleens of LMP7^−/−^ and C57BL/6 control mice 100 days p.i. (n = 3) and subjected to IFN-γ intracellular cytokine stain (IFN-γ ICCS) as for A.

Spleens from LMP7^−/−^ and C57BL/6 mice were examined 7 and 100 days p.i. using IFN-γ ICS to detect M38a-specific CD8^+^ T cells (Figure 4). During acute infection, an M38a-specific response was not detectable in LMP7^−/−^ mice compared to a small but readily detectable response (0.4%) in C57BL/6 mice (Figure 4A). By 100 days p.i., M38a specific responses were easily detectable in both C57BL/6 and LMP7^−/−^ mice (Figure 4B) although smaller in LMP7^−/−^ mice (2%) compared to C57BL/6 mice (5%). The increase in frequency of M38a-specific CD8^+^ T cells over time in C57BL/6 and LMP7^−/−^ mice indicated that memory inflation had occurred in both strains.

### Subdominant responses occur in LMP7^−/−^ mice but at reduced frequencies

**Figure 5:**
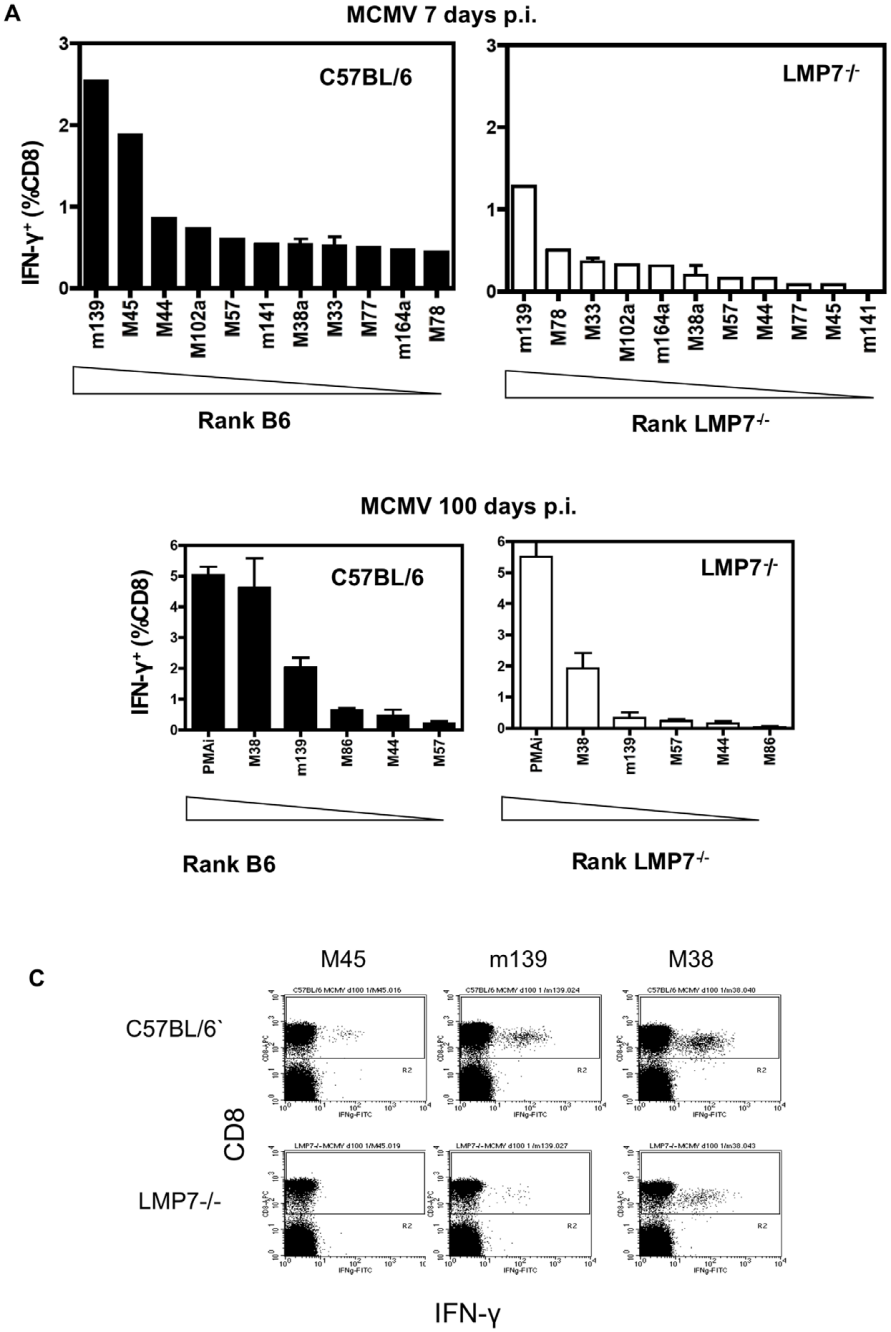
Breadth of CD8^+^ T cell responses to MCMV in LMP7^−/−^ mice. **A.** Splenocytes were prepared from spleens of LMP7^−/−^ and C57BL/6 control mice 7 days p.i. (n = 5), pooled, and exposed to each of nineteen synthetic peptides corresponding to defined CD8 epitopes of MCMV (Table S1), or media alone, in the presence of BFA for 5 hours. Splenocytes were subjected to CD8 surface stain, IFN-γ ICCS and FACS analysis as for Figure 1C. Frequency of IFN-γ cells among CD8^+^ T cells in infected C57BL/6 or LMP7^−/−^ mice, 7 days p.i. is shown for each peptide. Data is shown with background subtracted for both mouse strains, only for responses that in C57BL/6 mice were above background. Background is defined as three times the sum of the mean and standard error of the mean response to stimulation with media control alone. B. A similar analysis was to that described in Fig 5A was performed for CD8^+^ T cell responses detectable at 100 days p.i. C. Example ICCS staining for C57BL/6 and LMP7^−/−^ mice (day 100) in response to M45, m139 and M38 peptides.

Over 20 MCMV-derived CD8 epitopes have been defined in C57BL/6 mice [32]. It was possible that a previously subdominant epitope would be increased in LMP7^−/−^ mice, due to altered antigen processing or altered competition from other responses. As described above four immunodominant responses occurred at lower frequencies in LMP7^−/−^ mice relative to C57BL/6 mice (M45, m139, and M38a and m141). To analyze the other responses, splenocytes from LMP7^−/−^ and C57BL/6 mice, from day 7 and >50 p.i., were exposed to a panel of synthetic peptides corresponding to MCMV-derived CD8^+^ T cell epitopes previously defined in C57BL/6 mice (Table S1). Peptide-specific IFN-γ production by splenocytes from infected mice was assessed using ICS. In acute infection 10 out of 11 epitopes tested that stimulated responses in C57BL/6 mice also stimulated responses in splenocytes from acutely infected LMP7^−/−^ mice (Figure 5A). The frequency of 9 out of 11 responses was reduced relative to the equivalent responses in wild type mice (Figure 5A). Of the subdominant responses, two responses, to epitopes from M78 and M33, occurred at the same frequency in LMP7^−/−^ mice as in C57BL/6 mice, but overall these did not become immunodominant. The magnitude of all sub-dominant responses was low, close to the value of background for the assay.

At later timepoints 5 out of 5 epitopes that stimulated responses in C57BL/6 mice >50 days p.i. also stimulated responses in splenocytes from chronically infected LMP7^−/−^ mice (Figure 5B and C). All responses were reduced in frequency in LMP7^−/−^ mice relative to wild type responses.

### The M45 response is reduced in LMP7^−/−^ mice even when M45 is expressed by rVVs

**Figure 6:**
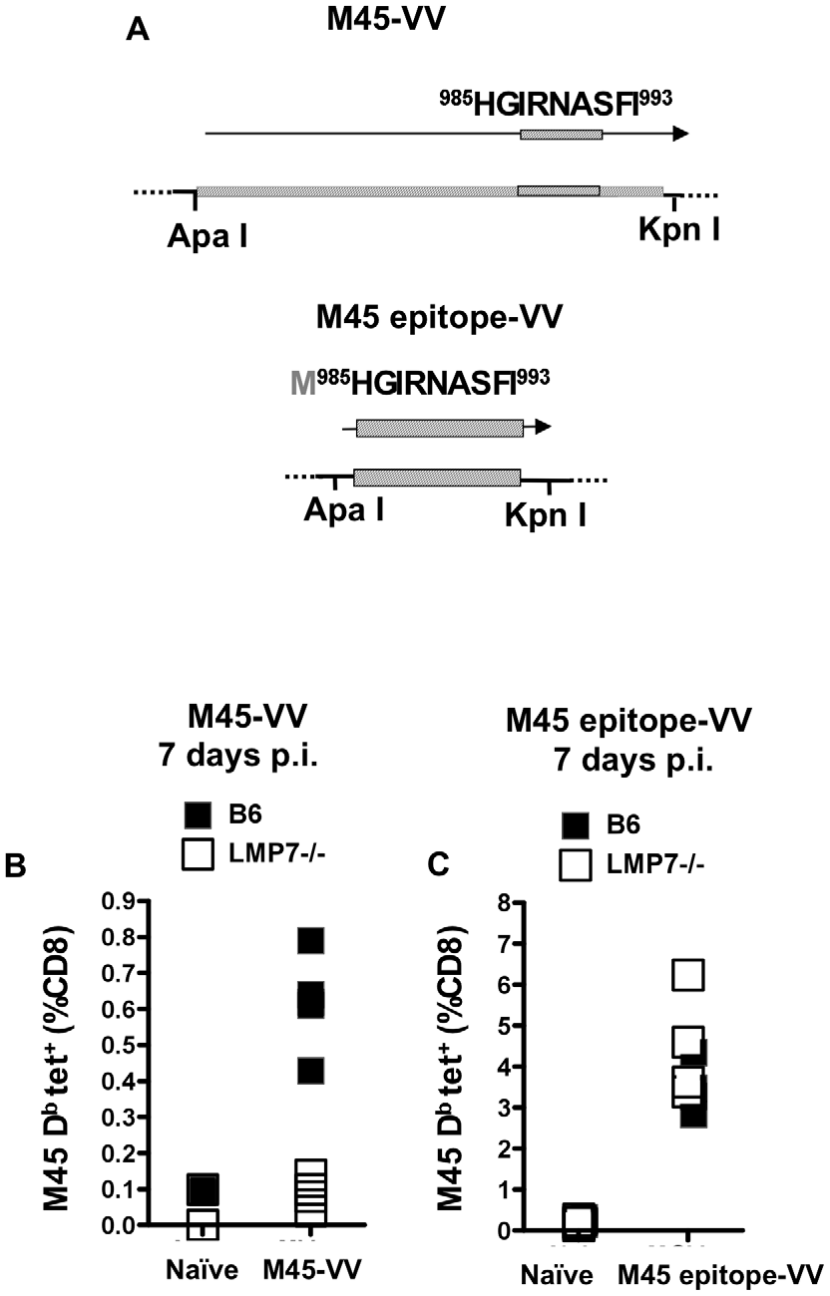
CD8^+^ T cell responses to M45 in LMP7^−/−^ mice infected with M45-expressing rVV. A. Recombinant vaccinia viruses (rVVs) were constructed containing foreign genes derived from the genomic sequence of MCMV Smith Strain (ATCC VR-194). Foreign genes were full length gene encoding the MCMV protein M45 (M45-VV), or a ‘minigene’, encoding the antigenic fragment of M45 corresponding to the CD8 epitope M45 H-2 D^b^ ^985^HGIRNASFI^993^ (M45 epitope-VV). Genes were designed for insertion between restriction sites for restriction endonucleases Apa I (5’) and Kpn I (3’). The diagram shows recombinant genes and proteins encoded by the two rVVs used in this study: M45 VV encoding full-length M45; and M45 epitope-VV, encoding the minimal D^b^-restricted CD8 epitope of M45 only. B, C. LMP7^−/−^ or C57BL/6 control mice were injected i.v. with 1×10^6^ pfu M45-VV (B), 1×10^6^ pfu M45 epitope-VV (C) or an equivalent volume of PBS. Blood was sampled 7 days p.i. by tail bleed. Peripheral blood lymphocytes were subjected to tetramer stain with M45 D^b^ HGIRNSFI tetramer and FACS analysis as for Figure 1A. The frequency of cells specific for M45 H-2 D^b^ ^985^HGIRNASFI^993^ among CD8^+^ T cells in the blood of MVacc and MGVacc - infected and mock-infected C57BL/6 and LMP7^−/−^ mice, 7 days after infection, is shown.

To determine whether loss of the CD8^+^ T cell response to M45 in LMP7^−/−^ was a quality associated with herpesvirus infection rather than a quality of the protein required expression of M45 protein in a different viral context to that in the native herpesvirus. Therefore, a novel rVV [34] expressing the M45 CD8^+^ T cell epitope as part of the full-length M45 protein (referred to as M45-VV), was constructed (Figure 6A). This was used to infect LMP7^−/−^ mice and assay for CD8^+^ T cell responses to M45.

C57BL/6 and LMP7^−/−^ mice were injected intravenously with M45-VV and 7 days p.i. peripheral blood was sampled. In C57BL/6 mice, 1% of CD8^+^ T cells were tetramer-positive (Figure 6B), while in contrast, peripheral blood from LMP7^−/−^ mice contained no M45-specific CD8^+^ T cells as determined by tetramer stains (Figure 6B). Thus the effect of LMP7 functional gene deletion on response to M45 was similar in LMP7^−/−^ mice infected with MCMV or with M45-VV.

We next sought to differentiate whether either (1) loss of immunoproteasome function or (2) a hole in the T cell repertoire might explain why the CD8^+^ T cells of LMP7^−/−^ mice failed to respond to M45 expressed from MCMV (Fig. 1) or M45-VV (Fig. 6B) [35]–[36]. To address this question, a second rVV was constructed that expressed the immunodominant 985–993 epitope of M45 from a minigene (M45 epitope-VV; Figure 6A and Table S2). C57BL/6 mice and LMP7^−/−^ mice were intravenously inoculated with M45 epitope-VV to determine if a CD8+ T cell response was mounted. On day 7 p.i. 4% of peripheral blood CD8^+^ T cells in both C57BL/6 mice and LMP7^−/−^ mice that received the M45 epitope-VV were specific for the M45 tetramer. We conclude that absence of M45-specific naïve T-cells (i.e. a hole in the repertoire) does not explain the failure of LMP7^−/−^ mice to respond to the M45 protein when expressed from MCMV or a vaccinia vector. Rather, the results imply that loss of the LMP7 protein compromises immunoproteasome function, which appears to be critical if the M45 is to be proteolytically processed such that the M45 immunodominant epitope is presented to CD8^+^ T cells.

## Discussion

In this study, we addressed the hypothesis that during MCMV infection CD8^+^ T cell responses specific for certain epitopes are better processed by the immunoproteasome than the constitutive proteasome. Immunoproteasomes and constitutive proteasomes produce differing sets of potentially antigenic peptides during MHC class I antigen processing for presentation to CD8^+^ T cells [21]. Professional APCs involved in priming naive CD8^+^ T cells constitutively express immunoproteasomes [18], [20]. In contrast, non-immune cells require exposure to IFN-γ to upregulate immunoproteasome expression [37]. During MCMV infection the viral protein M27 prevents IFN-γ-mediated upregulation of immunoproteasome expression in infected cells by blocking signaling through the IFNGR [30], [31]. It was possible that M27-mediated resistance to upregulation of immunoproteasome expression would protect infected cells from lysis by CD8^+^ T cell responses primed with constitutively immunoproteasome-rich cells [30]. This, however, is dependent on whether CD8^+^ T cell responses to MCMV are specific for epitopes better processed by the immunoproteasome than the constitutive proteasome (“immunoproteasome dependent”).

Using MCMV infection of LMP7^−/−^ mice and *ex vivo* CD8^+^ T cell assays for number and function, we found that the M45-specific CD8^+^ T cell response to MCMV that is immunodominant in C57BL/6 mice is profoundly affected by loss of immunoproteasomes. We also observed that most other responses tested that stimulated responses in C57BL/6 mice were reduced in LMP7^−/−^ mice. Since all MCMV-derived CD8^+^ T cell epitopes tested were affected by the loss of wild-type immunoproteasome in LMP7^−/−^ mice, and in studies of other viruses the effects on different epitopes from the same virus have been divergent [22], we speculated that the unusually consistent effect of the immunoproteasome was associated with herpesviral infection, and not specific qualities of the primary sequences of the proteins from which the epitopes were derived. Expressing the MCMV M45 (a protein containing the dominant CD8^+^ T cell epitope in C57BL/6 mice) in the context of rVV allowed testing of this. However, no response to M45 detectable directly *ex vivo* was induced in LMP7^−/−^ mice infected with rVV expressing full-length M45. The virus induced M45-specific responses in C57BL/6 mice, and LMP7^−/−^ mice produced M45-specific CD8^+^ T cell responses to rVV expressing only the M45 minimal epitope from a minigene. Therefore at least in the case of CD8^+^ T cell response to M45 the requirement for wild type immunoproteasome was maintained in the absence of MCMV infection. This suggested that M45 primary sequence and not viral interference determined the effect of the immunoproteasome on stimulation of M45-specific CD8^+^ T cell responses during MCMV infection. By extension, this suggests that MCMV-derived CD8^+^ T cell epitopes with responses reduced in LMP7^−/−^ mice might have protein primary sequences that could be better processed by immunoproteasome. Further work would be required to test this hypothesis more extensively; however, if true, these would be useful data points for immunoproteasome cleavage prediction. It should be pointed out that LMP7 deficient mice also lack a contribution from LMP2 and MECL in immunoproteasomes [38], so it remains open which of these subunits is actually required for the generation of the different epitopes.

These findings have a number of implications for viral pathogenesis. Firstly it is known that M45-specific CD8^+^ T cell populations have limited protective capacity in a bone marrow transplant model [33], [39]. This can be potentially explained by the fact while these responses may be readily generated in LMP7^+^ DCs, for example through cross-presentation [40], the target cells themselves, which are low in immunoproteasomes, will present only inefficiently. Thus the relative imbalance in immunoproteasome distribution between APCs and target cells may have special relevance in MCMV infection. Other viruses where IFN-γ signaling blockade also occurs [41] might show similar phenomena.

The generalized impact of LMP7 depletion on the pattern of CD8^+^ T cell responses to MCMV epitopes was also interesting. A number of MCMV epitopes have been mapped, although many of the CD8^+^ T cell responses are at relatively low levels compared to the well-defined M45 response focused upon in these studies [32]. A caveat of this study is the accuracy of measurement of changes in these very small populations. However, it is evident that a shift in immunodominance towards these subdominant responses did not occur. We did not in this study measure the overall impact of LMP7 depletion on viral load over time. However, a number of previous studies in C57BL/6 mice in which specific deletion of CD8^+^ T cells has been performed [7], [42], or in CD8 gene knockout [42], [43] or MHC Class I deficient mice [44], did not show an impact of this single intervention so a substantial effect would not be expected. The impact of CD8^+^ T cell depletion may be substantially different in other mouse strains, such as BALB/c, where the role of protective NK cells is less evident [43]. Studies using novel immunoproteasome inhibitors [28], or using mutant viruses which resist NK cells through deletion [45] or mutation in M157 [46] may also provide insight into the impact of immunoproteasome-dependent CD8^+^ T cell populations on viral load. Alternatively there may be important but subtle effects on viral reactivation and latency as evidenced by experiments in the susceptible BALB/c strain where single responses have been deleted through viral mutation [47].

Notably responses to M38 and m139 are sustained or increase over time compared to classical “memory” responses, a phenomenon described as memory “inflation”. This feature was first described in the BALB/c (susceptible) model [13], [14], but subsequently observed in the C57BL/6 (resistant) model [11] using natural and transgenic epitopes. Re-exposure of such “inflating” populations to antigen over time appears to underlie generation of these CD8^+^ T cell pools [2], a hypothesis supported by the “effector memory” phenotypic characteristics detectable in the cell subsets [11], [12]. One reason proposed why specific epitopes attract “inflating” populations compared to others, is the generation of peptides that escape the viral mechanisms for downregulation of antigen processing. In particular, IE-1 derived peptides might generate inflating populations since they are generated early after infection [14]. However, this is not uniquely the case as epitopes from later expressed proteins can also be associated with inflation [13], [32] and there are situations where distinct peptides may be generated from the same protein (M38) in which one epitope is inflating while the other is not [11]. We note that the two inflating epitopes included in this study (M38 and m139) had the least dependence on immunoproteasomes. We speculate that this feature could contribute to their unique immunologic profile. During chronic infection, in addition to the constraints placed upon antigen presentation by viral gene expression kinetics and immunoevasins, the ability to be presented by cells low in LMP7 may provide an additional “filter” to limit the number of inflating epitopes.

While the M38 CD8^+^ T cell response does show inflation over time, the m139 response, whilst still dominant, is maintained stably and does not substantially further increase over time. This feature has been noted previously in some MCMV responses [11]; some responses are clearly maintained by antigen exposure (and thus do not revert to “central” memory), but show long-term stability rather than increase. In this case the overall size of the population may be somewhat reduced compared to wild-type response due to a limitation in antigen presentation, or alternatively by competition with newly arising responses.

The question of whether other novel CD8^+^ T cell responses arise is an important one. As a marker of overall T cell memory, there was not a deficit in the proportion of splenic CD8^+^ T cells responding to PMA/Ionomycin through IFN-γ secretion in LMP7 deficient mice at day 100 (data not shown). Thus it is possible that responses outside those measured are entering the memory pool. Further detailed analyses of novel specificities is required to define any new responses, which may potentially normally be limited by immunoproteasomes [19].

The responses analyzed in this paper remain functional, as measured here by IFN-γ secretion, although not all cells demonstrated using tetramer staining were detectable in short term functional assays (e.g. Fig 1). Overall, when this has been extensively analyzed [11], [12], [14] these responses are both functional (in terms of cytokine secretion, killing and proliferation) and protective, and in contrast to LCMV there is no evidence of exhaustion. Given recent studies of the impact of LMP7 inhibition on T cell derived IL-17 in tissue, it would be interesting to explore this specific aspect in future in MCMV [48].

**Figure 7:**
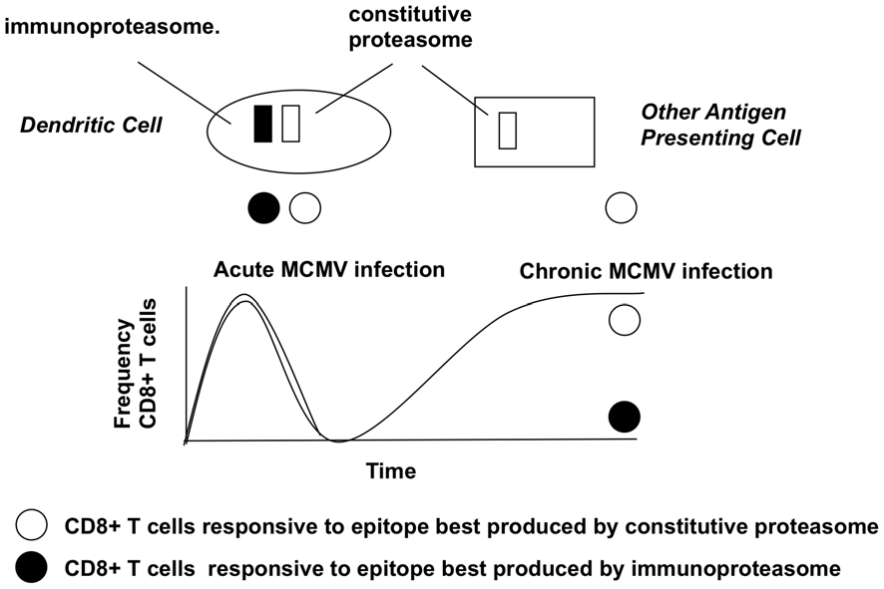
“2-cell” model to explain different effect of LMP7 on “inflating” memory and classical memory CD8^+^ T cell responses to MCMV. Infected or cross-presenting DCs during acute infection contain immunoproteasomes and constitutive proteasomes and stimulate responses to epitopes produced by either proteasome. In chronic infection other APCs containing only constitutive proteasomes stimulate responses only to those epitopes produced by the constitutive proteasome resulting in selection of more immunoproteasome-independent responses during chronic infection.

Combining the data we therefore propose a “2-cell” model for the role of the immunoproteasome in MCMV (Figure 7). Under normal circumstances in acute disease, antigen may be presented or cross-presented on DCs and prime strong responses: since DCs constitutively express immunoproteasomes, and the responses are highly immunoproteasome dependent, a wide range of such responses will be generated. Such responses are, however, unable to protect infected cells against infection if these cells downregulate LMP7. As infection is controlled, antigen production is limited by a variety of factors, including NK cells, but in LMP7^lo^ cells, LMP7 dependent epitopes will not be re-presented and classical memory will ensue. Epitopes that show some independence from LMP7 will have the potential to be re-presented and from this limited pool, “inflating” epitopes are selected. Since DCs are constitutive expressors of LMP7, this suggests that the cells responsible for antigen presentation during chronic infection could include other APCs or infected cell populations. More generally, this data suggests that DC-based strategies which prime CD8^+^ T cell responses in an immunoproteasome-rich environment may run the risk of selecting CD8^+^ T cell populations which are unable to recognize target cells, in settings where a virus (or even tumor) has modified its immunoproteasome cargo.

Overall our findings suggest that LMP7 and the immunoproteasome play a more significant role in MCMV than previously suspected from studies of other virus infections. This may be relevant to human HCMV infection and potentially infection with other viruses which display comparable dynamics. To what extent this situation has evolved to provide a viral advantage is not clear, but the extent of the impact is very evident, and appears to be a property of the viral sequence itself. These data can be added to the renewed accumulation of interest in the impact of the immunoproteasome in infection and inflammation, following studies on LCMV [28] and toxoplasma [49] (recently reviewed in Ref. [50]). Further experiments to explore the role of the immunoproteasome in herpesviral infections will be of great interest in the future.

## Methods

Ethics statement: Mouse experiments were performed according to UK Home Office regulations (project licence number PPL 30/2235) and after review and approval by the local ethical review board at the University of Oxford.

### Mice and viruses

C57BL/6 and LMP7^−/−^ mice were bred in a specific pathogen free animal facility unit at the John Radcliffe Hospital, University of Oxford, United Kingdom. MCMV (Strain Smith, ATCC: VR-194) was provided by Professor U.H. Koszinowski, Department of Virology, Max von Pettenkofer Institute, Munich, Germany. Vaccinia virus Western Reserve (VVWR, ATCC: VR-1354) was used. Mice were injected intravenously (i.v.) with 1×10^6^ pfu (100 µl) MCMV or rVV as indicated.

#### Peptides

Peptide stock solutions (1 mM) (Roswell Park Memorial Institute media 1640 (RPMI; Sigma), di-methyl sulfoxide (DMSO) (10%)) were stored at −80°C before use then at 4°C for up to 2 months. DMSO concentrations in final cell assays were less than 0.01%. Synthetic peptides with amino acid sequences matching CD8^+^ T cell epitopes from MCMV (Table S1) were custom synthesized to 80–85% purity (Weatherall Institute for Molecular Medicine peptide synthesis facility, John Radcliffe Hospital, University of Oxford).

#### Staining protocols

R10 (RPMI-1640, foetal calf serum (FCS) (10%), PSG (penicillin (5,000 U/ml) streptomycin (5 mg/ml) and glutamine (5 mM)), β-mercaptoethanol (β-ME) (50 mM)) was used for temporary storage of lymphocytes after removal of red blood cells (RBC) and of splenocytes before and during preparation from whole spleens. FACSWash (PBS, FCS (2%), EDTA (5 mM)) was used to suspend lymphocytes during staining for FACS analysis. FACS Fix (phosphate buffered saline (PBS), Para-formaldehyde (PFA) (1%)) was used to fix lymphocytes after staining and prior to FACS analysis. Biotinylated MHC class I molecules refolded with human β-2 microglobulin (β2M) and peptide were stored in PBS at −80°C before use. Antibodies (Ab) for fluorescence activated cell sorting (FACS) analysis included CD8-PerCP or CD8-APC (Invitrogen). Intracellular stains used IFN-γ FITC after stimulation as previously described. All flow cytometry was performed and analyzed using CellQuest3.3 or FloJo acquisition software and FACScalibur flow cytometer (BD Biosciences).

#### Generation of rVVs

MCMV M45 gene sequence data was derived from NCBI sequence database, accession: ‘MuHV1_gpM45’, NCBI GeneID: 3293809. Primers for amplification and cloning of MCMV gene M45 were Forward primer: aagggcccaccATGGATCGCCAGCCCAAAGTC; Reverse primer: taggtaccTCAGCGATAATTCACGGAAGGGG). BioX-Act Long Mix (Bioline) was used for PCR. For the minigene, complementary oligo-nucleotides (oligos) were designed such that when annealed they form a single short fragment of dsDNA or insert encoding the amino acid sequence of the MHC class I epitope M^985^HGIRNASFI^993^ (Sense strand: caccATGCACGGCATCAGGAACGCCTCCTTCATCTGAggtac; Antisense strand: cTCAGATGAAGGAGGCGTTCCTGATGCCGTGCATggtgggcc). For cloning, Apa I, Kpn I, Sma I and Xmn I restriction enzymes and T4 ligase were obtained from New England Biolabs (NEB). Bacteria competent for transformation used in cloning were TOP10 (Invitrogen), used for amplification of plasmids less than 12 kilobase pairs (kbp) in length or XL-1 Blue (Stratagene) used for plasmids greater than 12 kbp.

All rVVs were prepared using previously described protocols [34] and were confirmed by sequencing. For minigene recombinants two primers were used: (Forward: CAAACCCACCCGCTTTTTAT; Reverse: TACGTTGAAATGTCCCATCG). Sequencing full-length M45 gene required 16 additional primers (Table S2). rVV prepared from viral plaques was subjected to PCR using an ‘inside’ primer specific for a sequence unique to the recombinant M45 full-length gene (GCGCCGCGGCCGCTCGGCG) or M45 D^b^ ^985^HGIRNASFI^993^ minigene (GATGAAGGAGGCGTTCCTGATGCCGTGC) as appropriate. Sequence Scanner (v1.0, Applied Biosystems) was used to determine quality of sequence data. Sequence data was searched for mismatches with published sequence using Clustal W (EMBL). Vaccinia viruses were grown in thymidine kinase-negative human osteosarcoma fibroblast cell line TK-143 (ATCC: CRL-8303).

## Supporting Information

Table S1 Amino acid sequences of MCMV CD8+ T cell epitopes referred to in this study.
(0.11 MB DOCX)

Table S2 Primers used for sequencing full-length gene M45.
(0.12 MB DOCX)
