# Supplementary figures and images for: A Dominant Role for the Immunoproteasome in CD8+ T Cell Responses to Murine Cytomegalovirus
Source: PLoS One. 2011 Feb 3;6(2):e14646. doi: 10.1371/journal.pone.0014646 (PMC3033404; doi:10.1371/journal.pone.0014646)

**Table S1.** Amino acid sequences of MCMV CD8 epitopes referred to in this study.


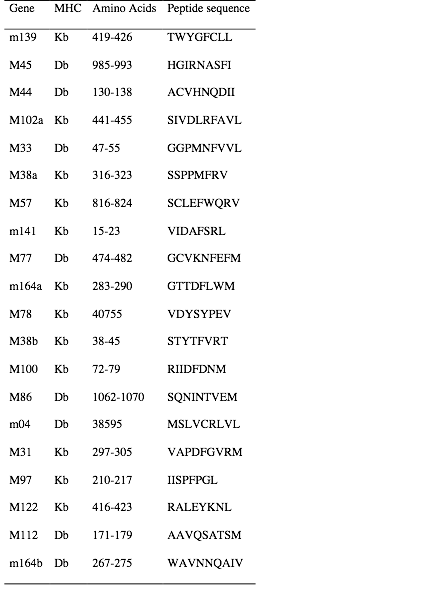

Supplement: Table S1 — Amino acid sequences of MCMV CD8+ T cell epitopes referred to in this study. (0.11 MB DOCX) [file pone.0014646.s001.docx]

**Table S2.** Primers used for sequencing full-length gene M45.


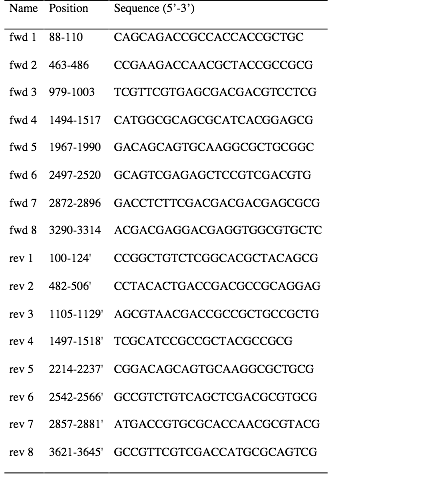

Supplement: Table S2 — Primers used for sequencing full-length gene M45. (0.12 MB DOCX) [file pone.0014646.s002.docx]
